# The incidence of subclinical atherosclerosis in subjects with low and moderate cardiovascular risk

**DOI:** 10.1002/clc.24087

**Published:** 2023-07-31

**Authors:** Yan Li, Yinghua Zhang, Keling Xiao, Jin Si, Haoyu Zhang, Lijie Sun, Zupei Miao, Ting Zhao, Jinghao Sun, Xipeng Sun, Zhi Liu, Jing Gao, Jing Zhao, Xi Chu, Jing Li

**Affiliations:** ^1^ Department of Geriatrics, Xuanwu Hospital, Capital Medical University National Clinical Research Center for Geriatric Diseases Beijing China; ^2^ Department of Cardiology Chui Yang Liu Hospital affiliated to Tsinghua University Beijing China; ^3^ Department of Cardiology, Xuanwu Hospital Capital Medical University Beijing China; ^4^ Health Management Center, Xuanwu hospital Capital Medical University Beijing China

**Keywords:** brachial flow‐mediated dilation, brachial‐ankle pulse wave velocity, China‐par risk model, Framingham risk model, subclinical atherosclerosis

## Abstract

**Background:**

The cardiovascular risk models and subclinical atherosclerotic indicators are both recommended for cardiovascular risk stratification. The accordance between the incidence of subclinical atherosclerosis and subjects with low and moderate cardiovascular risk is unclear.

**Hypothesis:**

Subjects with low and moderate cardiovascular risk have a lower incidence of subclinical atherosclerosis compared with subjects with high risk.

**Methods:**

Brachial‐ankle pulse wave velocity (BaPWV) and brachial flow‐mediated dilation (BFMD) were measured in 421 subjects without a history of atherosclerotic cardiovascular disease (ASCVD) from October 2016 to January 2020. All subjects were classified into low, moderate, and high risk based on Framingham and China‐par risk models respectively.

**Results:**

Mean age was 57.05 ± 9.35 years and 248 (58.9%) were male. In subjects with low, moderate, and high risk assessed by Framingham and China‐par risk models, the percentage of abnormal BaPWV ( > 1400 cm/s) was 42.9%, 70.1%, 85.7%, and 40.4%, 71.4%, 89.7%, respectively. Meanwhile, the percentage of abnormal BFMD ( ≤ 7%) was 43.8%, 68.5%, 77.3%, and 44.9%,72.1%, and 76.6%. According to Framingham‐based high‐risk categories, positive predictive value (PPV), negative predictive value (NPV), sensitivity and specificity for BaPWV abnormality were 85.7%, 39.4%, 36.1%, and 87.5%, respectively. For BFMD abnormality, the values were 77.3%, 40.1%, 34.1%, and 81.8%, respectively. According to China‐par high‐risk categories, the values for BaPWV abnormality were 89.7%, 43.8%, 45.6%, and 89.0%, respectively. For BFMD abnormality, the values were 76.6%, 41.3%, 40.7%, and 77%, respectively. In multivariate analysis, age and blood pressure were the independent predictors for subclinical atherosclerosis in subjects with low‐moderate risk.

**Conclusions:**

More than one‐half of subjects with low and moderate risk already have detectable subclinical atherosclerosis, indicating higher cardiovascular risk beyond the traditional stratification.

## Graphical abstract

The relationship between subclinical atherosclerotic parameters and cardiovascular risk groups, including Framingham risk groups and China‐par risk groups. About 40% of low‐risk populations and 70% of moderate‐risk populations already had subclinical atherosclerosis based on Framingham and China‐par risk groups, respectively.

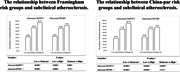

## 1 INTRODUCTION

Atherosclerotic cardiovascular disease (ASCVD) is the leading cause of death and disease burden worldwide.^1^, ^2^, ^3^ To prevent ASCVD, risk stratification models have been recommended in the current guidelines to guide the primary prevention.^4^, ^5^ For example, the Framingham Heart Study developed the first ASCVD risk model based on traditional risk factors in the American population in 1976,^6^, ^7^ which has been widely used in primary prevention. Recently, the China‐par risk model has been validated in the prediction for 10‐year ASCVD risk in Chinese population.^8^ Nevertheless, some studies suggest that the performance of current risk models may not be satisfied in identifying ASCVD risk for those with low and moderate risk.^9^, ^10^, ^11^ For example, Murphy et al. found that 75%–87% of myocardial infarction (MI) or coronary death occurred in populations with low and moderate risk.^9^ Brindle and Orford reported that about 40% of major adverse cardiovascular and cerebrovascular events (MACCEs) occurred in individuals with Framingham model‐based low and moderate risk.^10^, ^11^

In recent years, some subclinical atherosclerotic indicators, reflecting either structural or functional damage in the arterial wall, have been demonstrated to effectively predict future cardiovascular risk in individuals without prior history of ASCVD. For example, a meta‐analysis showed that arterial stiffness, expressed as pulse wave velocity (PWV), predicts the incidence of future cardiovascular disease (CVD), and improves risk classification.^12^ Moreover, the abnormal flow‐mediated dilation (FMD), which reflects endothelial dysfunction, may present higher cardiovascular risk in the future.^13^ Therefore, a series of subclinical atherosclerotic indicators have been used to improve the accuracy of risk stratification.^5^, ^14^

In this study, subjects without ASCVD were classified into low‐, moderate‐, and high‐risk groups by the Framingham and China‐par risk models separately. PWV and FMD were measured as subclinical atherosclerotic parameters. We aim to investigate the accordance between the incidence of subclinical atherosclerosis and subjects with low and moderate cardiovascular risk.

## 2 METHODS

### 2.1 Study population

Subjects, who had measurements of brachial‐ankle pulse wave velocity (BaPWV), and brachial flow‐mediated dilation (BFMD) in Health Management Center, Xuanwu hospital Capital Medical University from October 2016 to January 2020, were included in the current study. Subjects were excluded if (1) subjects were younger than 30 years old or older than 74 years old; (2) subjects had a history of MI, percutaneous coronary intervention, ischemic stroke, hemorrhagic stroke, transient ischemic attack which were acquired by subjects’ medical records; (3) lumen diameter stenosis is higher than 50% in coronary artery as determined by coronary angiography (CAG); (4) lumen diameter stenosis is higher than 50% in intracranial artery as determined by transcranial color doppler ultrasound (TCCD); (5) lumen diameter stenosis is higher than 50% in peripheral artery as determined by peripheral arterial ultrasonography. Informed consent forms were signed by all subjects.

### 2.2 Data collection

We collected demographic characteristics (including age, sex, body mass index [BMI], waist circumference, state of smoking and history of diseases [family history of ASCVD, hypertension, diabetes mellitus]), systolic blood pressure (SBP), diastolic blood pressure (DBP), laboratory results (blood glucose, blood uric acid, total cholesterol [TC], triglyceride, high‐density lipoprotein cholesterol [HDL‐C], low‐density lipoprotein cholesterol [LDL‐C]) and subclinical atherosclerotic parameters (BaPWV and BFMD) from subjects’ medical records and questionnaires. Laboratory examinations were conducted when subjects were fasting for at least 8 h.

### 2.3 Measurement of BaPWV

We measured BaPWV by a volume plethysmographic method using automatic waveform analyzer. Before measurements, patients were required to be in the supine position and rest for at least 5 min. Cuffs were wrapped around both upper arms and ankles. BaPWV was calculated by measuring the time for the pulse wave to travel between the brachial and posterior tibial arteries.^15^ The maximum value of the left and right BaPWV was used in the statistical analyses. BaPWV＞1400 cm/s was regarded as abnormal BaPWV.^16^

### 2.4 Measurement of BFMD

The subjects remained supine throughout the study. Vasodilator responses in the brachial arteries were measured using B‐mode ultrasound images with a 7.5‐MHz linear array transducer. The brachial artery was scanned in the antecubital fossa in a longitudinal fashion. After baseline measurements of the diameter and flow velocity in the brachial artery, a blood pressure (BP) cuff was placed around the forearm and inflated to a pressure of 250–300 mmHg for 5 min, and then released. Diameter measurements during reactive hyperemia were taken 45–90 s after cuff deflation. The changes in vessel diameter in response to reactive hyperemia (FMD) were expressed as a percentage increase in diameter from the baseline value.^17^ BFMD ≤ 7% was seen as abnormal BFMD.^18^

### 2.5 Calculation of ASCVD risk scores

Framingham general CVD risk model was designed for 10‐year risk prediction about ASCVD events including coronary death, MI, coronary insufficiency, angina, ischemic stroke, hemorrhagic stroke, transient ischemic attack, peripheral artery disease, heart failure. Risk categories were defined as low‐risk ( ≤ 6%), moderate‐risk (6%–20%), and high‐risk ( > 20%) groups according to the Framingham Heart Study.^7^ China‐par risk model evaluated ASCVD (nonfatal acute MI or coronary heart disease death or fatal or nonfatal stroke) events during the next 10 years. Risk categories were defined as low‐risk ( < 5%), medium‐risk (5%–9.9%), and high‐risk ( ≥ 10%) groups. Online calculators for Framingham and China‐par risk models are available at https://framinghamheartstudy.org/fhs-risk-functions/cardiovascular-disease-10-year-risk/ and https://www.cvdrisk.com.cn/ASCVD/Eval.

### 2.6 Statistical analysis

Continuous variables were described as mean ± standard deviation or median (interquartile range), and differences among the groups were assessed by the independent *t* test or the Wilcoxon rank‐sum test. Categorical variables were described as number (n) with percentage (%), and differences were analyzed by the *χ* ^2^ test or Fisher exact test. For subclinical atherosclerosis, the sensitivity and specificity of ASCVD risk models were evaluated by receiver operating characteristic curve  analysis. The relationship between subclinical atherosclerosis and risk factors was evaluated by multivariable logistic regression analysis. We included the following covariates in the multivariate analysis: age, gender, SBP, DBP, hypertension, smoking, diabetes mellitus, TC, HDL‐C. SPSS version 22.0 (IBM Corp.) was used for statistical analysis, and a *p* value < .05 was defined as the threshold of statistical significance.

## 3 RESULTS

From October 2016 to January 2020, 874 subjects measured BaPWV and BFMD in Department of Cardiology and Department of Health Management, Xuanwu hospital Capital Medical University. Four hundred and fifty‐three subjects were excluded due to prior history of ASCVD. Four hundred and twenty‐one individuals were eligible for analysis. The mean age of subjects were 57.05 ± 9.35 years, and 248 (58.9%) were male. There were 114 (27.1%), 187 (44.4%), and 68 (16.2%) had smoking, hypertension, and diabetes. Mean BMI values of subjects were 25.77 ± 3.72 kg/m^2^. Among 421 subjects, the mean values of Framingham risk models were 15.77 ± 13.02%. There were 105 (24.9%), 197 (46.8%), and 119 (28.3%) subjects had low‐, moderate‐, and high‐risk of 10‐year ASCVD events according to the Framingham risk model. The mean values of China‐par risk models were 8.57 ± 5.80%. There were 136 (32.3%), 140 (33.3%), and 145 (34.4%) subjects had low‐, moderate‐, and high‐risk of 10‐year ASCVD events according to the China‐par risk model. The mean BaPWV and BFMD of 421 subjects were 1510.86 ± 256.28 cm/s and 6.23 ± 2.99%. There were 285 (67.7%) and 273 (64.8%) subjects had abnormal BaPWV ( > 1400.0 cm/s) and BFMD ( ≤ 7.0%) among included participants. The clinical characteristics of these subjects are listed in Table 1.

**Table 1:** Clinical characteristics of subjects.

Variables	*N* = 421
Age (years)	57.05 ± 9.35
Males, *n* (%)	248 (58.9)
BMI (kg/m^2^)	25.77 ± 3.72
Waist circumference (cm)	89.29 ± 10.39
Current smoker, *n* (%)	114 (27.1%)
SBP (mmHg)	129.13 ± 15.96
DBP (mmHg)	78.13 ± 11.35
Family history of ASCVD, *n* (%)	53 (12.6%)
Peripheral vascular disease, *n* (%)	7 (1.7)
Hypertension, *n* (%)	187 (44.4%)
Diabetes mellitus, *n* (%)	68 (16.2%)
*Laboratory results*	
Serum glucose (mmol/L)	5.29 [4.90, 5.96]
Serum uric acid (µmol/L)	347.79 ± 97.18
TC (mmol/L)	4.47 ± 1.01
Triglyceride (mmol/L)	1.47 [1.09, 2.12]
HDL‐C (mmol/L)	1.25 ± 0.36
LDL‐C (mmol/L)	2.72 ± 0.83
*Parameters of subclinical atherosclerosis*	
BaPWV (cm/s)	1510.86 ± 256.28
BaPWV abnormality, *n* (%)	285 (67.7)
BFMD (%)	6.23 ± 2.99
BFMD abnormality, *n* (%)	273 (64.8)
*ASCVD risk models*	
Framingham risk score (%)	15.77 ± 13.02
Low‐risk group ( ≤ 6%), *n* (%)	105 (24.9)
Moderate‐risk group (6% to 20%), *n* (%)	197 (46.8)
High‐risk group ( > 20%), *n* (%)	119 (28.3)
China‐par risk score (%)	8.57 ± 5.80
Low‐risk group ( < 5%), *n* (%)	136 (32.3)
Moderate‐risk group (5% to 9.9%), *n* (%)	140 (33.3)
High‐risk group ( ≥ 10%), *n* (%)	145 (34.4)

Abbreviations: ASCVD, nonfatal acute myocardial infarction or coronary heart disease death or fatal or nonfatal stroke; BaPWV, brachial‐ankle pulse wave velocity; BFMD, brachial flow‐mediated dilation; BMI, body mass index; DBP, diastolic blood pressure; HDL‐C, high‐density lipoprotein cholesterol; LDL‐C, low‐density lipoprotein cholesterol; SBP, systolic blood pressure; TC, total cholesterol.

**Figure 1:**
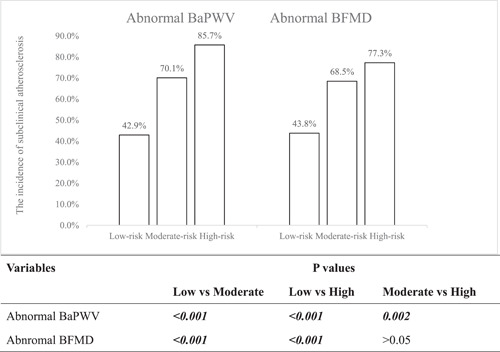
The relationship between subclinical atherosclerotic parameters and Framingham risk groups.

**Figure 2:**
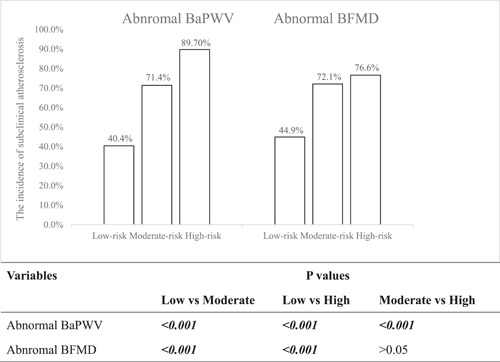
The relationship between subclinical atherosclerotic parameters and China‐par risk groups.

The relationship between Framingham‐based groups and subclinical atherosclerosis was shown in Figure 1. Among the low‐risk group, there were 45 (42.9%) and 46 (43.8%) subjects had BaPWV and BFMD abnormality, respectively. Among the moderate‐risk group, there were 138 (70.1%) and 135 (68.5%) subjects had BaPWV and BFMD abnormality, respectively. Among the high‐risk group, there were 102 (85.7%) and 92 (77.3%) subjects had BaPWV and BFMD abnormality, respectively. The relationship between China‐par risk groups and subclinical atherosclerosis was shown in Figure 2. Among the low‐risk group, there were 55 (40.4%) and 61 (44.9%) subjects had BaPWV and BFMD abnormality separately. Among the moderate‐risk group, there were 100 (71.4%) and 101 (72.1%) subjects had BaPWV and BFMD abnormality separately. Among the high‐risk group, there were 130 (89.7%) and 111 (76.6%) subjects had BaPWV and BFMD abnormality separately.

According to Framingham risk models, positive predictive value (PPV) for BaPWV abnormality was 85.7% in subjects with high risk, negative predictive value (NPV) was 39.4% in low‐ to moderate‐risk population, sensitivity was 36.1%, and specificity was 87.5%. For BFMD abnormality, PPV, NPV, sensitivity, and specificity were 77.3%, 40.1%, 34.1%, and 81.8%, respectively. According to China‐par risk categories, PPV, NPV, sensitivity, and specificity for BaPWV abnormality were 89.7%, 43.8%, 45.6%, and 89.0%, respectively. For BFMD abnormality, PPV, NPV, sensitivity, and specificity were 76.6%, 41.3%, 40.7%, and 77%, respectively (Table 2).

**Table 2:** The accuracy of subclinical atherosclerosis among low‐ to moderate‐risk group and high‐risk group.

	Framingham risk groups		China‐par risk groups	
	BaPWV abnormality (%)	BFMD abnormality (%)	BaPWV abnormality (%)	BFMD abnormality (%)
Sensitivity	36.1	34.1	45.6	40.7
Specificity	87.5	81.8	89.0	77.0
NPV	39.4	40.1	43.8	41.3
PPV	85.7	77.3	89.7	76.6

Abbreviations: BaPWV, brachial‐ankle pulse wave velocity; BFMD, brachial flow‐mediated dilation; NPV, negative predictive value; PPV, positive predictive value.

According to the Framingham risk model, age (odds ratio [OR]: 1.093, 95% confidence interval [CI]: 1.059–1.128, *p* < .001) and SBP (OR: 1.076, 95% CI: 1.052–1.100, *p* < .001) were independent risk factors for BaPWV abnormality in low‐ to moderate‐risk group. For BFMD abnormality, age (OR: 1.060, 95% CI: 1.030–1.091, *p* < .001), DBP (OR: 1.088, 95% CI: 1.043–1.135, *p* < .001), and hypertension (OR: 1.789, 95% CI: 1.009–3.172, *p* < .001) were independent risk factors. When divided by the China‐par risk model, age (OR: 1.090, 95% CI: 1.054–1.127, *p* < .001) and SBP (OR: 1.069, 95% CI: 1.044–1.094, *p* < .001) were independent risk factors for abnormal BaPWV in low‐ to moderate‐risk group. For abnormal BFMD, age (OR: 1.056, 95% CI: 1.023–1.089, *p* = .001), DBP (OR: 1.036, 95% CI: 1.008–1.064, *p* = .011), and hypertension (OR: 2.065, 95% CI: 1.154–3.696, *p* = .015) were independent risk factors (Table 3). The clinical characteristics of low‐ to moderate‐risk population divided by BaPWV and BFMD abnormality are listed in Supporting Information: Table 1‐4.

**Table 3:** Relationship between subclinical atherosclerosis with risk factors in low‐ to moderate‐risk population.

Variables	Abnormal BaPWV			Abnormal BFMD		
	OR	95%CI	*p* values	OR	95%CI	*p* values
*Subjects with low‐ to moderate‐risk assigned by Framingham risk model*						
Age	1.093	(1.059, 1.128)	<.001	1.060	(1.030, 1.091)	<.001
SBP	1.076	(1.052, 1.100)	.001	‐		.106
DBP	‐		.681	1.088	(1.043, 1.135)	<.001
Hypertension	‐		.830	1.789	(1.009, 3.172)	.046
Males	‐		.072	‐		.634
Current smoker	‐		.413	‐		.921
Diabetes mellitus	‐		.263	‐		.990
TC	‐		.586	‐		.567
HDL‐C	‐		.748	‐		.311
*Subjects with low‐ to moderate‐risk assigned by China‐par risk model*						
Age	1.090	(1.054, 1.127)	<.001	1.056	(1.023, 1.089)	.001
SBP	1.069	(1.044, 1.094)	<.001	‐		.329
DBP	‐		.157	1.036	(1.008, 1.064)	.011
Hypertension	‐		.749	2.065	(1.154, 3.696)	.015
Males	‐		.218	‐		.141
Current smoker	‐		.626	‐		.448
Diabetes mellitus	‐		.530	‐		.619
TC	‐		.799	‐		.793
HDL‐C	‐		.815	‐		.395

Abbreviations: BaPWV, Brachial‐ankle pulse wave velocity; BFMD, Brachial flow‐mediated dilation; CI, confidence interval; DBP, diastolic blood pressure; HDL‐C, high density lipoprotein cholesterol; OR, odds ratio; SBP, systolic blood pressure; TC, total cholesterol.

In our study, 307 subjects have completed the vascular imaging tests. We further divided these populations into two groups, 119 subjects with <50% stenosis atherosclerosis and 188 subjects with no evidence of atherosclerosis. Compared with populations without stenosis, participants with <50% stenosis atherosclerosis have higher incidence of abnormal BaWPV (83.19% vs. 63.83%, *p* < .001) and BFMD (78.15% vs. 63.30%, *p* = .006). (Supporting Information: Figure 1).

## 4 DISCUSSION

In this study, we evaluated ASCVD risks through risk models and measured BaPWV and BFMD in each risk category. In the high‐risk group, the majority of subjects (76.6‐89.7%) had abnormal BaPWV and BFMD. More importantly, the abnormal BaPWV and BFMD also appeared in 40.4%–44.9% of the low‐risk group and 68.5%–72.1% of the moderate‐risk group, indicating the existence of structural or functional damage in the artery. We also found age and BP were independent predictors for abnormal BaPWV and BFMD in the low‐ to moderate‐risk group.

ASCVD risk models, such as Framingham and China‐par risk models, have been recommended in the current guidelines to predict the risk of future cardiovascular events and guide the primary prevention.^4^, ^5^ However, previous studies indicated that the performance of current risk models might not be satisfactory in identifying cardiovascular risk, especially for subjects with low‐ and moderate risk.^9^, ^10^, ^11^ Orford et al. found the Framingham risk model underestimated the incidence of cardiovascular events in the very low‐risk group ( < 5%).^11^ By comparing observed events rates with events rates predicted by the Framingham risk model in 1700 subjects, the Whickham (UK) study found the two rates were similar in the high‐risk population, but the events rates were underestimated by the Framingham risk model in those at low‐ and moderate‐risk populations.^19^ Moreover, recent studies investigated the relationship between predicted cardiovascular risk and subclinical atherosclerotic injury.^20^, ^21^ According to Eleid's study, 43% of subjects in the low‐risk group and 62% of subjects in the moderate‐risk group had carotid plaque or thickening of carotid intima‐media.^20^ Canpolat et al. also found that 33.8% of subjects with low‐risk scores had coronary atherosclerotic plaque.^21^ In our study, Framingham and China‐par risk models were used to predict cardiovascular risk, and the abnormal BaPWV and BFMD, reflecting arterial stiffness and endothelial dysfunction separately, were defined as subclinical atherosclerosis. The majority of subjects in the high‐risk group had abnormal BaPWV and BFMD. Whereas more than half of the subjects in the low‐ to moderate‐risk group also had subclinical atherosclerosis. Previous studies have demonstrated that abnormal BaPWV was closely associated with twofold risk of coronary heart disease and stroke,^16^ and per percent point decreasing in BFMD indicated a significant 10% higher risk of cardiovascular events.^22^ Hence, the low‐ and moderate‐risk populations with subclinical atherosclerosis may have higher cardiovascular risk than that initially assessed by ASCVD risk models.

The reasons for the poor performance of ASCVD risk models in subjects with low‐ and moderate risk are still uncertain. Generally, risk models were developed based on traditional risk factors, including age, gender, waist circumference, diabetes, smoking, BP, TC, and HDL cholesterol. It is likely that the existence of nontraditional risk factors, such as inflammatory and metabolic abnormality, may contribute to subclinical atherosclerotic injury and subsequent higher risk of cardiovascular events.^23^ For example, higher levels of high sensitivity C‐reactive protein (hsCRP) and Lipoprotein‐associated phospholipase A2 (Lp‐PLA2), biomarkers of inflammation, are associated with both subclinical atherosclerosis and increased incidence of major cardiovascular events, independent of traditional risk factors.^24^, ^25^, ^26^ High level of lipoprotein (a) (Lp[a]), an indicator of remnant metabolic risk, is an independent predictor for subclinical coronary atherosclerosis^27^ and is associated with a 48% increased risk of MI after adjustment for traditional risk factors.^28^ In addition, risk scores are modifiable. The scores vary with the changes in traditional risk factors, such as BP, blood lipid, and other parameters. A single risk score only reflects the cardiovascular risk at that time, but cannot reveal the dynamic changes of cardiovascular risk. Whereas subclinical atherosclerosis directly reflects the injury of comprehensive risk factors on the artery wall. Previous studies have demonstrated that subclinical atherosclerosis was closely associated with the higher risk of future ASCVD events.^16^, ^22^ Thus, the combination of ASCVD risk models and subclinical atherosclerotic parameters may be more accurate to predict cardiovascular risk than applying ASCVD risk models alone.

The risk models were used to classify populations into low‐, moderate‐, and high‐risk groups in current guidelines. Then, the primary prevention strategies were offered respectively.^5^, ^14^ Intensive interventions are required for high‐risk populations to reduce cardiovascular risk. For low‐ to moderate‐risk subjects, routine prevention strategies were appropriate to maintain their low‐ to moderate‐risk status.^5^ However, for those low‐ and moderate‐risk populations with subclinical atherosclerosis, intensive interventions may also be needed to slow, halt, or even reverse the progression of atherosclerotic injury.^29^ Takamitsu et al.^30^ showed that after achieving the optimal goals of traditional risk factors, subjects with improvement in either BaPWV or BFMD alone tended to reduce 69% risk for cardiovascular events, and subjects with improvement in both BaPWV and BFMD had 86% reduced risks. Therefore, it may be reasonable to combine ASCVD risk models and subclinical atherosclerotic parameters to guide prevention strategies, especially for subjects with low and moderate cardiovascular risk.

According to our findings, age and BP were independent predictors for subclinical atherosclerosis in low‐ to moderate‐risk population after adjustment for various risk factors. Tomiyama et al. included 12 517 subjects to evaluate the influences of age on BaPWV and the results were consistent with our study.^31^ In addition, our study found that low levels of BP were closely associated with low incidence of subclinical atherosclerosis. The systolic blood pressure intervention trial study^32^ and the strategy of blood pressure intervention in the elderly hypertensive patients) trial^33^ have demonstrated that targeting lower levels of systolic BP resulted in lower rates of MACCEs. Future studies and more evidence are needed to determine whether controlling BP strictly can reduce the risk of subclinical atherosclerosis.

In our study, more than half of the populations with no evidence of atherosclerosis, evaluated by vascular imaging tests, already have abnormal BaPWV or BFMD. Populations with abnormal BaPWV and BFMD had a higher risk of cardiovascular events in the future.^12^, ^13^ Thus, those people, who had abnormal BaPWV and BFMD, may need more strict prevention strategies, even if they did not have <50% stenosis atherosclerosis.

### 4.1 Limitations

The current study examines the incidence of subclinical atherosclerosis in subjects with low and moderate cardiovascular risk assessed by ASCVD risk models. However, this study has some limitations. First, this was a single‐center observational study, which may lead to data bias. Second, we do not have follow‐up data for subclinical atherosclerosis. Thus, we are not sure whether intensive interventions can improve subclinical atherosclerotic indicators. Third, subclinical atherosclerosis includes various parameters, such as BaPWV, BFMD, coronary artery calcium (CAC), and carotid plaque. Our study only analyzed the BaPWV and BFMD. Fourth, 114 of the included subjects did not finish the vascular imaging tests, and we were not sure whether these subjects have <50% stenosis atherosclerosis.

## 5 CONCLUSIONS

Considerable subjects with low‐ to moderate cardiovascular risk already have abnormal BaPWV and BFMD, indicating higher cardiovascular risk beyond the stratification based on ASCVD risk models. The combination of ASCVD risk models and subclinical atherosclerotic parameters may be more accurate to predict cardiovascular risk and guide prevention strategies than applying ASCVD risk models alone.

## Supporting information

Supporting information.

## Data Availability

The data that support the findings of this study are available on request from the corresponding author. The data are not publicly available due to privacy or ethical restrictions.
