# Supplementary material for: The incidence of subclinical atherosclerosis in subjects with low and moderate cardiovascular risk
Source: Clin Cardiol. 2023 Jul 31;46(10):1260–7. doi: 10.1002/clc.24087 (PMC10577528; doi:10.1002/clc.24087)
Supplement: Supplementary file 1 — Supporting information. [file CLC-46-1260-s001.docx]

**Supplementary Table 1 Clinical characteristics of Framingham low- to moderate-risk population divided by BaPWV abnormality**

| **Variables** | **Normal BaPWV**  **N=119** | **Abnormal BaPWV**  **N=183** | **P values** |
| --- | --- | --- | --- |
| Age (years) | 50.85±8.86 | 56.14±7.90 | <0.001 |
| Males, n(%) | 68 (57.1) | 73 (39.9) | 0.003 |
| BMI (kg/m^2^) | 25.42±3.82 | 25.24±3.65 | 0.688 |
| Waist circumference (cm) | 87.42±10.50 | 87.62±11.14 | 0.879 |
| Current smoker, n(%) | 27 (22.7) | 21 (11.5) | 0.009 |
| SBP (mmHg) | 117.77±11.96 | 126.65±12.41 | <0.001 |
| DBP (mmHg) | 71.94±9.00 | 77.44±9.95 | <0.001 |
| Family history of ASCVD, n(%) | 12 (10.1) | 27 (14.8) | 0.237 |
| Peripheral vascular disease, n (%) | 1 (0.9) | 5 (2.9) | 0.447 |
| Hypertension, n(%) | 30 (25.2) | 85 (46.4) | <0.001 |
| Diabetes mellitus, n(%) | 4 (3.4) | 20 (10.9) | 0.031 |
| ***Laboratory results*** | |  |  |
| Blood glucose (mmol/l) | 5.13 [4.84, 5.67] | 5.29 [4.90, 5.75] | 0.028 |
| Blood uric acid (umol/l) | 349.19±96.62 | 341.14±100.76 | 0.503 |
| TC (mmol/l) | 4.61±0.87 | 4.45±1.00 | 0.169 |
| Triglyceride (mmol/l) | 1.40 [0.98, 2.00] | 1.43 [1.04, 1.92] | 0.531 |
| HDL-C (mmol/l) | 1.33±0.40 | 1.29±0.39 | 0.393 |
| LDL-C (mmol/l) | 2.82±0.72 | 2.69±0.83 | 0.179 |

BaPWV, brachial-ankle pulse wave velocity; BMI, Body Mass Index; SBP, systolic blood pressure; DBP, diastolic blood pressure; ASCVD, nonfatal acute myocardial infarction or coronary heart disease death or fatal or nonfatal stroke; TC, total cholesterol; HDL-C, high density lipoprotein cholesterol; LDL-C, low density lipoprotein cholesterol.

**Supplementary Table 2 Clinical characteristics of Framingham low- to moderate-risk population divided by BFMD abnormality**

| **Variables** | **Normal BFMD**  **N=121** | **Abnormal BFMD**  **N=181** | **P values** |
| --- | --- | --- | --- |
| Age (years) | 52.36±9.29 | 56.59±9.15 | <0.001 |
| Males, n(%) | 56 (46.3) | 85 (47.0) | 0.908 |
| BMI (kg/m^2^) | 25.23±3.97 | 25.69±3.53 | 0.293 |
| Waist circumference (cm) | 86.72±11.88 | 88.69±9.71 | 0.129 |
| Current smoker, n(%) | 22 (18.2) | 26 (14.4) | 0.374 |
| SBP (mmHg) | 123.12±14.88 | 127.04±13.44 | 0.018 |
| DBP (mmHg) | 73.07±9.61 | 77.80±10.48 | <0.001 |
| Family history of ASCVD, n(%) | 15 (12.4) | 24 (13.3) | 0.827 |
| Peripheral vascular disease, n (%) | 3 (2.5) | 3 (1.7) | 0.950 |
| Hypertension, n(%) | 32 (26.4) | 83 (45.9) | 0.001 |
| Diabetes mellitus, n(%) | 8 (6.6) | 16 (8.8) | 0.483 |
| ***Laboratory results*** | |  |  |
| Blood glucose (mmol/l) | 5.15 [4.79, 5.51] | 5.31 [4.91, 5.78] | 0.082 |
| Blood uric acid (umol/l) | 336.80±99.60 | 342.02±96.63 | 0.650 |
| TC (mmol/l) | 4.58±0.88 | 4.44±1.05 | 0.233 |
| Triglyceride (mmol/l) | 1.34 [0.93, 1.93] | 1.47 [1.09, 2.05] | 0.022 |
| HDL-C (mmol/l) | 1.38±0.44 | 1.28±0.34 | 0.043 |
| LDL-C (mmol/l) | 2.78±0.74 | 2.69±0.87 | 0.319 |

BFMD, Brachial flow-mediated dilation; BMI, Body Mass Index; SBP, systolic blood pressure; DBP, diastolic blood pressure; ASCVD, nonfatal acute myocardial infarction or coronary heart disease death or fatal or nonfatal stroke; TC, total cholesterol; HDL-C, high density lipoprotein cholesterol; LDL-C, low density lipoprotein cholesterol.

**Supplementary Table 3 Clinical characteristics of China-par low- to moderate- risk population divided by BaPWV abnormality**

| **Variables** | **Normal BaPWV**  **N=121** | **Abnormal BaPWV**  **N=155** | **P values** |
| --- | --- | --- | --- |
| Age (years) | 50.86±9.03 | 57.52±8.73 | <0.001 |
| Males, n(%) | 74 (61.2) | 82 (52.9) | 0.17 |
| BMI (kg/m^2^) | 25.35±3.75 | 25.60±3.69 | 0.578 |
| Waist circumference (cm) | 87.24±10.43 | 88.33±10.81 | 0.386 |
| Current smoker, n(%) | 34 (28.1) | 35 (22.6) | 0.293 |
| SBP (mmHg) | 118.35±12.39 | 130.10±13.29 | <0.001 |
| DBP (mmHg) | 72.25±9.33 | 78.28±10.37 | <0.001 |
| Family history of ASCVD, n(%) | 9 (7.4) | 22 (14.2) | 0.078 |
| Peripheral vascular disease, n (%) | 0 (0) | 2 (1.4%) | 0.504 |
| Hypertension, n(%) | 29 (24.0%) | 62 (40.0%) | 0.005 |
| Diabetes mellitus, n(%) | 4 (3.3) | 11 (7.1) | 0.267 |
| ***Laboratory results*** | |  |  |
| Blood glucose (mmol/l) | 5.12 [4.84, 5.49] | 5.23 [4.85, 5.85] | 0.199 |
| Blood uric acid (umol/l) | 342.32±99.97 | 338.38±96.44 | 0.732 |
| TC (mmol/l) | 4.63±0.87 | 4.41±1.05 | 0.052 |
| Triglyceride (mmol/l) | 1.35 [0.94, 1.94] | 1.48 [1.14, 2.10] | 0.259 |
| HDL-C (mmol/l) | 1.35±0.40 | 1.30±0.38 | 0.275 |
| LDL-C (mmol/l) | 2.83±0.73 | 2.66±0.87 | 0.073 |

BaPWV, brachial-ankle pulse wave velocity; BMI, Body Mass Index; SBP, systolic blood pressure; DBP, diastolic blood pressure; ASCVD, nonfatal acute myocardial infarction or coronary heart disease death or fatal or nonfatal stroke; TC, total cholesterol; HDL-C, high density lipoprotein cholesterol; LDL-C, low density lipoprotein cholesterol.

**Supplementary Table 4 Clinical characteristics of China-par low- to moderate-risk population divided by BFMD abnormality**

| **Variables** | **Normal BFMD**  **N=114** | **Abnormal BFMD**  **N=162** | **P values** |
| --- | --- | --- | --- |
| Age (years) | 51.46±8.35 | 55.48±8.62 | <0.001 |
| Males, n(%) | 58 (50.9) | 98 (60.5) | 0.113 |
| BMI (kg/m^2^) | 25.12±3.94 | 25.46±3.56 | 0.466 |
| Waist circumference (cm) | 86.60±11.98 | 88.18±9.95 | 0.250 |
| Current smoker, n(%) | 25 (21.9) | 44 (27.2) | 0.323 |
| SBP (mmHg) | 121.68±13.91 | 123.51±12.26 | 0.250 |
| DBP (mmHg) | 73.24±9.86 | 76.29±9.78 | 0.011 |
| Family history of ASCVD, n(%) | 13 (11.4) | 18 (11.1) | 0.940 |
| Peripheral vascular disease, n (%) | 1 (0.9) | 1 (0.6) | 1.000 |
| Hypertension, n(%) | 23 (20.2) | 68 (42.0) | <0.001 |
| Diabetes mellitus, n(%) | 6 (5.3) | 9 (5.6) | 0.916 |
| ***Laboratory results*** | |  |  |
| Blood glucose (mmol/l) | 5.16 [4.79, 5.58] | 5.18 [4.88, 5.66] | 0.803 |
| Blood uric acid (umol/l) | 347.76±99.90 | 342.49±98.39 | 0.664 |
| TC (mmol/l) | 4.63±0.89 | 4.45±0.99 | 0.118 |
| Triglyceride (mmol/l) | 1.35 [0.93, 1.95] | 1.48 [1.10, 2.07] | 0.037 |
| HDL-C (mmol/l) | 1.37±0.44 | 1.27±0.35 | 0.041 |
| LDL-C (mmol/l) | 2.82±0.74 | 2.70±0.81 | 0.200 |

BFMD, Brachial flow-mediated dilation; BMI, Body Mass Index; SBP, systolic blood pressure; DBP, diastolic blood pressure; ASCVD, nonfatal acute myocardial infarction or coronary heart disease death or fatal or nonfatal stroke; TC, total cholesterol; HDL-C, high density lipoprotein cholesterol; LDL-C, low density lipoprotein cholesterol.

**Supplementary Figure 1 Comparison of subclinical atherosclerotic parameters in subjects with no evidence of atherosclerosis and subjects with <50% stenosis atherosclerosis**

| **Variables** | **No stenosis (188)** | **<50% stenosis (119)** | **P values** |
| --- | --- | --- | --- |
| Abnormal BaPWV | 120 (63.83%) | 99 (83.19%) | ***<0.001*** |
| Abnromal BFMD | 119 (63.30%) | 93 (78.15%) | ***0.006*** |

The percentages of populations with subclinical atherosclerosis are presented on each bar; BaPWV, Brachial-ankle pulse wave velocity; BFMD, Brachial flow-mediated dilation.
